# Brain regions involved in moxibustion-induced analgesia in irritable bowel syndrome with diarrhea: a functional magnetic resonance imaging study

**DOI:** 10.1186/1472-6882-14-500

**Published:** 2014-12-16

**Authors:** Yi Zhu, Zhiyuan Wu, Xiaopeng Ma, Huirong Liu, Chunhui Bao, Ling Yang, Yunhua Cui, Cili Zhou, Xiaomei Wang, Yuemin Wang, Zhongwei Zhang, Huan Zhang, Haipeng Jia, Huangan Wu

**Affiliations:** Shanghai Research Institute of Acupuncture and Meridian, Shanghai University of Traditional Chinese Medicine, No.650 South Wanping Road, Shanghai, 200030 P.R. of China; Department of Radiology, Ruijin Hospital, Shanghai Jiao Tong University School of Medicine, No. 197 Ruijin Er Road, Shanghai, 200025 China; Shanghai Community Health Service Center of Shi Men Er Road, No. 456 Shi Men Er Road, Shanghai, 200041 China

**Keywords:** Moxibustion, fMRI, D-IBS

## Abstract

**Background:**

Moxibustion is one of the most commonly used therapies in acupuncture practice, and is demonstrated to be beneficial for patients with diarrhea from irritable bowel syndrome (D-IBS). But its mechanism remains unclear. Because visceral hypersensitivity in IBS patients has been documented by evaluation of perceived stimulations through functional magnetic resonance imaging (fMRI) studies, we focused on observing brain imaging changes in D-IBS patients during rectal balloon distention before and after moxibustion in order to reveal its possible central mechanism and further evaluate its effect.

**Methods:**

This clinical trial is registered under the number: ChiCTR-TRC-10000887. Eighty D-IBS patients were randomly divided into a moxibustion and sham moxibustion group (control group) for a 4-week treatment. Fifteen patients in moxibustion group and thirteen patients in control group completed two fMRI scans during a 50 and 100 ml rectal balloon distention before and after treatment. Rectal pain were obtained with a scan test. Birmingham IBS Symptom Scale and IBS Quality of Life (QOL) Scale were used to evaluate therapeutic effect.

**Results:**

After treatment, the decrease in Birmingham IBS Symptom Scale and IBS QOL Scale scores in moxibustion group was significantly greater than that of control group (P < 0.01). The defecation urge threshold and the pain perception threshold of moxibustion group was also significantly higher after treatment than that of control group (P < 0.01). The decrease in pain score during the 100 ml rectal balloon distention in moxibustion group was significantly greater than that of control group (P < 0.05). There was no definite activated center during the 50 ml rectal distention in either group before treatment. After treatment, the prefrontal cortex (PFC) was affected in moxibustion group, while the PFC and the anterior cingulated cortex (ACC) were affected in control group. During the 100 ml distention before treatment in both groups, the PFC and ACC were activated. After treatment, they disappeared in moxibustion group but remained in control group.

**Conclusions:**

Moxibustion can improve symptoms and quality of life in D-IBS patients. It can also decrease rectal sensitivity. The activation of PFC and ACC during a 100 ml rectal distention disappeared after moxibustion treatment.

## Background

Irritable bowel syndrome (IBS) is a lasting condition involving recurrent attacks of a group of clinical symptoms including abdominal pain, distention, bowel habit changes, and abnormal stool. It is a common gastrointestinal (GI) disorder, affecting 10–15% of the population in developed countries. The global prevalence rate of IBS is 11.2% [1] the prevalence in western countries is from 4.7% to 25% while it’s from 6.5% to 10.1% in eastern countries [2]. In China, it’s about 4.6%-5.67% [3]. Among IBS patients in China, 74.1% of them are diarrhea predominant IBS(D-IBS), which is the most common type[4]. Traditional Chinese medicine has long been used to treat IBS. A Meta-analysis showed acupuncture exhibits clinically and statistically significant controls of IBS symptoms [5]. Studies showed that in comparative effectiveness Chinese trials, patients reported greater benefits from acupuncture than from pharmacological therapies [6, 7]. One study finding s indicate that Acu/Moxa treatment shows promise in the area of symptom management for IBS [8]. In fact, moxibustion treatment for D-IBS has been shown to have a good clinical effect [9, 10]. It can effectively relieve diarrhea, abdominal pain, abdominal distension, and other symptoms [11]. Chen Sheng et al. found that moxibustion treatment had better results than Pinaverium [12]. Although the risk of bias in some included studies is relatively high, one systematic review and meta-analysis suggests that moxibustion may provide benefits to IBS patients [13]. Guangqing An et al. found that acupuncture–moxibustion is more effective than medication for treating IBS. Moreover, moxibustion is often more easily accepted by patients [14]. Therefore, moxibustion helps D-IBS patients improve their symptoms, although its mechanism remains unclear.

In recent years, the visceral hypersensitivity of IBS patients has received much attention. Visceral hypersensitivity relates to stress, emotions, and the brain–gut axis [15–17]. Increased sensitivity of the rectum is seen in almost all IBS patients, especially from stimulation, such as rectal balloons or mechanical stimulation. Visceral sensitivity, rectal sensory thresholds, and compliance of patients with D-IBS were found to be significantly higher than those in control patients [18–20]. Weak stimulation, which does not result in a response from normal patients, can produce the perception of IBS. Equal subliminal stimulations cause a greater response in IBS patients than those in control patients [21].

Rapid developments in functional brain imaging have led to the visual observation of central visceral pain, and activities in areas of the brain. A recent meta-analysis of published studies on brain responses to rectal distension supports the conclusion that brain responses to rectal distension differ between IBS patients and healthy controls(HCs) [22]. Moreover, cerebral cortex blood flow, glucose metabolic rate, and potential activities of IBS are not the same as those in control groups [23, 24]. Although the results of brain imaging studies on IBS are not entirely consistent, they suggest that visceral hypersensitivity in IBS patients could relate to nerve centers, and especially the dysregulation of the pain nerve center.

Moxibustion can effectively relieve the symptoms of D-IBS such as diarrhea, abdominal pain, and abdominal discomfort. However, further assessments are needed to explain the therapeutic effects because there are no studies fully explaining the central mechanisms of moxibustion. The aim of this study was to examine changes in the brain imaging of D-IBS patients via functional magnetic resonance imaging (fMRI) during rectal balloon distention before and after moxibustion to understand its central mechanism and further evaluate its clinical effect.

## Methods

### Participants

Eighty right-handed patients were recruited from the outpatient clinic of Shanghai Research Institute of Acupuncture and Meridian and the Shanghai Community Health Service Center of Shi Men Er Road from April 2010 to November 2011. Each patient met the Rome III criteria of D-IBS. The enrolled patients were randomly divided into a moxibustion group (n = 40) or a sham moxibustion group (control group) (n = 40) using a computer-generated randomization sequence. The sequence was concealed from the care providers through the use of sealed, opaque, sequentially numbered envelopes. Patients were blinded to group assignment. Exclusion criteria in both groups included patients with: clinically relevant gastrointestinal, hepatic, or other systemic diseases; bowel resections or abdominal operations; any medication administered in preceding 30 days; pregnancy or lactation; or epilepsy.

**Figure 1:**
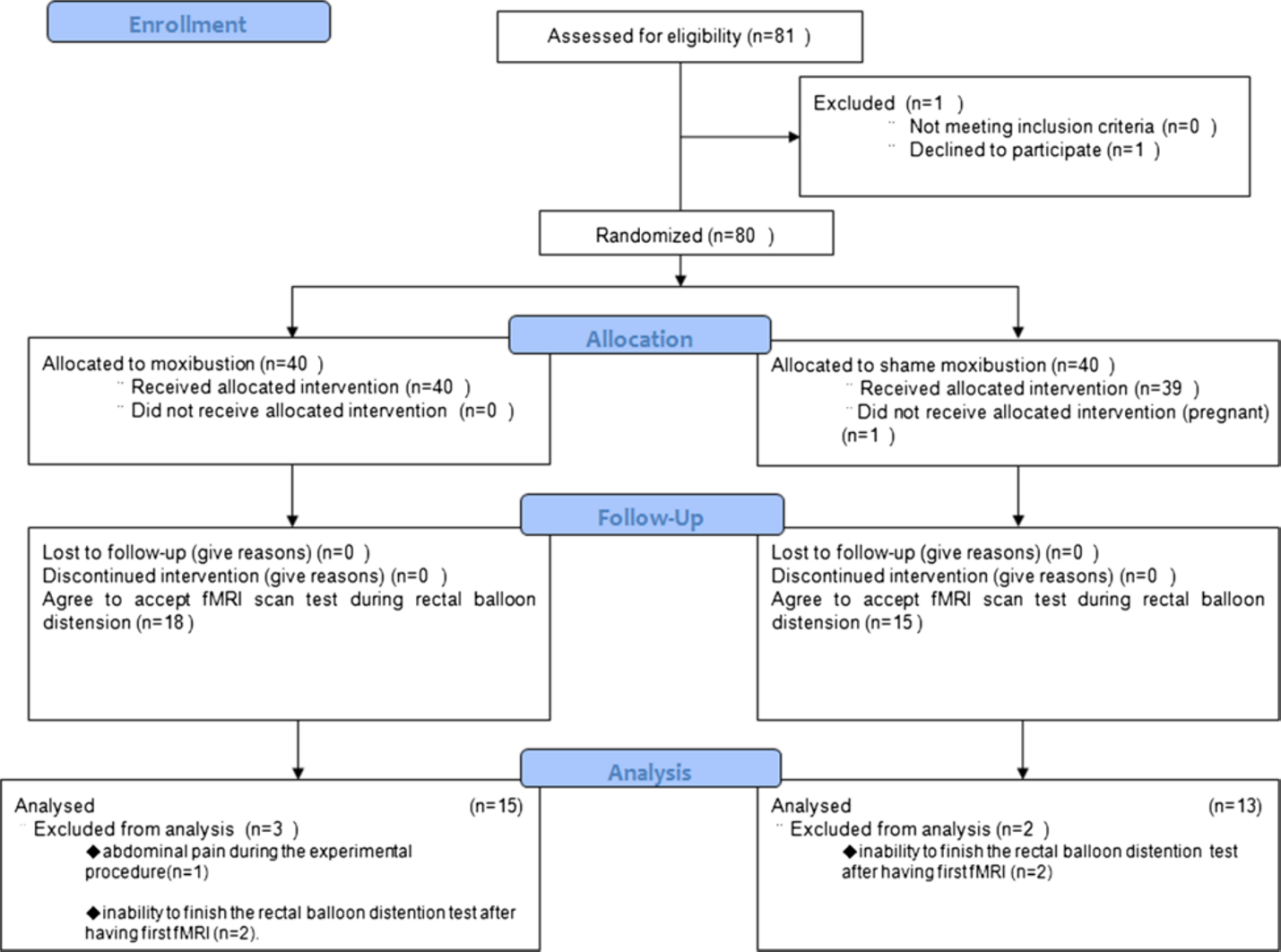
**The consort flow chart.**

Forty patients in the moxibustion group and 39 patients in control group finished the treatment intervention. One patient in the control group dropped out after several treatments because of pregnancy. After explaining the fMRI procedure to the included patients, 46 patients declined the rectal balloon distension. Thirty-three patients agreed to have the fMRI test. Among them, 18 patients were in the moxibustion group and 15 were in the control group. Fifteen patients in the moxibustion group completed two fMRI scans. Three patients in the moxibustion group withdrew from the study. Reasons for discontinuation included: abdominal pain during the experimental procedure (n = 1), and the inability to finish the rectal balloon distention test after having the first fMRI (n = 2). Thirteen patients in the control group completed two fMRI scans. Two patients in the control group dropped out from an inability to finish the rectal balloon distention test. Overall, 15 patients in moxibustion group (six females, nine males) (mean 47.5 ± 0.896 years, range 41–53) and 13 patients in control group (six females, seven males) (mean 40.9 ± 10.136 years, range 35–47) finished the study. The flow chart of the clinical trials is shown in Figure 1.

The clinical trial number of this study is: ChiCTR-TRC-10000887. The study protocol was approved by Ethics Committee of Shanghai traditional Chinese Medicine University affiliated Yue Yang hospital of Integrated Traditional Chinese and Western medicine, Shanghai University of Traditional Chinese Medicine in March 2010 (Authorization No. 2010–01; issue date: 3rd of March 2010). Written informed consent was obtained from each participant.

### Treatment

**Figure 2:**
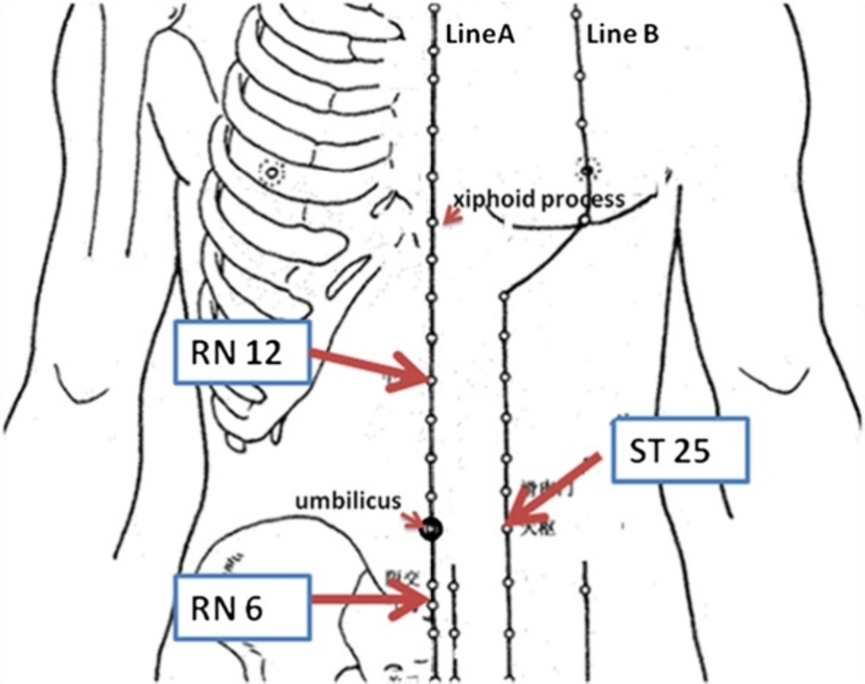
**Locations of acupoint (LineA: RenMeridian, LineB: Stomach Meridian).** The acupoints were located as follows: (i) ST25(Tianshu), 2 cun lateral to the centre of the umbilicus; (ii)Ren12(Zhongwan), on the middle of the abdomen, 4 cun above the umbilicus; (iii)Ren6(Qihai), on the midline of the abdomen,1.5cun below the umbilicus. It’s 8 cun from xiphoid process to the center of the umbilicus and 5cun between the center of the umbilicus and the upper border of symphysis pubis.

Acupoints Tianshu (ST25), Qihai (RN6), and Zhongwan (RN12) were used in the moxibustion group. Aconite cakes (specially molded with a diameter of 2.5 cm, height of 1 cm, and weight of 5.8 g) were put on the above points and burning moxa (from Nanyang, China, moxa cone of 1.5 cm in diameter, 1.5 cm in height, and 1.6 g in weight) were placed on the cakes. One moxa cone was used for each treatment, three times per week for two weeks as a course of treatment. Every patient had two courses of treatment. In the control group, the same aconite cakes and moxa cones were used. However, round cardboard pieces (2 cm in diameter, 1.14 g in weight) were placed under the aconite cakes. The treatment course was the same as that of the moxibustion group. The locations of the acupoints are shown in Figure 2.

### Clinical assessments

The Birmingham IBS Symptom Scale was used to rate patient symptoms. The score includes multiple dimensions that cover 14 representative and relevant questions of IBS [25]. Symptoms are graded on a six point scale: none of the time (0 point), a little of the time (1 point), some of the time (2 points), a good bit of the time (3 points), most of the time (4 points), all of the time (5 points). The IBS QOL Scale [26] was used to compare the quality of life of patients. The scale includes 34 items and the subscale structures are: dysphoria, interference with activity, body image, health worry, food avoidance, social reaction, sexual, and relationship. The scores are divided into five levels: not at all (1 point), slightly (2 points), moderately (3 points), quite a bit (4 points), and extremely or a great deal (5 points). All scores from these two scales for patients in both groups before and after treatment were recorded. Lower scores indicate milder symptoms and better QOL.

### Statistical analysis

#### Sample size

Because there was no reference to indicate the effect size that could be expected from the use of moxibustion to treat IBS with diahhra, we did not estimate the sample size based on a power calculation. Instead, we enrolled 80 participants with a 20% withdrawal rate to provide 32 patients in each group in order to meet the number more than that of the requirement of minimum sample size.

#### Clinical variables

The scores of the Birmingham IBS Symptom Scale and IBS QOL Scale were analyzed with SPSS 18.0. Repeated measure was used for statistical comparison.

### Brain fMRI scans

#### Stimulation

Rectal stimulation was performed by distending a custom-designed polyethylene balloon (50 mm in length, 20 mm in diameter, and a maximum volume of 320 ml, Hefei Austrian Bio-technology Co. Ltd, Hefei, China). The balloon was attached to a plastic injector via a 30-cm-long tube. The balloon could be inflated with air from the injector. Rectal balloon distention stimulation on the rectum was presented in separate functional runs conducted during two different sessions with the stimulus order counter balanced across sessions. For each subject, the balloon catheter was passed perianally and positioned in the rectum 10 to 15 cm above the anus at the start of the visceral experiment. The stimulation sequences were identical, consisting of two stimulus intensities: “high”, which distended the balloon with 100 ml of air, and “low,” which distended the balloon with 50 ml of air.

Each patient was scanned by fMRI under rectal balloon distention before and after treatment. Each test subject was recorded before the injection of gas for different feeling intensities, including initial feeling thresholds, urgent defecation perception threshold, and pain perception threshold. Then, the rectal pain score (Visual analog scale: 0 = none to 10 = unbearable) of the subjects during balloon gas injection to 50 ml or 100 ml was recorded. These recorded data were analyzed with SPSS 18.0 and the paired samples test was used for statistical comparison.

#### Imaging procedure

MRI was performed using a 1.5 T GE Scanner (Exicte HD, General Electric Medical System, Milwaukee, WI, USA) with an 8-channel NVHEAD coil. Each session consisted of one anatomical scan and two functional scanning runs. The anatomical scans were recorded using a high-resolution T1-weighted anatomical protocol (TR 8.1 ms, TE 60.0 ms, slice thickness 1.4 mm, FOV 24 cm × 18 cm). The functional scans were collected using a blood-oxygen-level-dependent (BOLD) protocol with a T2*-weighted gradient echo-planar imaging (EPI) sequence (TR 3000 ms, TE 60 ms, flip angle 90°). The scanning planes were oriented parallel to the anterior commissure-posterior commissure line and covered the whole brain from the base of the cerebellum to the top of the cortex (32 slices, slice thickness 5 mm). The individual scans consisted of 60 whole brain volume acquisitions, divided into three cycles. Each cycle consisted of 30 s (ten successive volume acquisitions) with visceral stimulation, followed by 30 s without stimulation. Extra baseline (12 s) with no stimulation was added in the beginning of each scanning run. So the total scan includes: simulation stage (30 s), resting stage (30 s), simulation stage (30 s), resting stage (30 s), simulation stage (30 s), resting stage (30 s), totally 180 s. Before being positioned in the scanner, all subjects were instructed to attend to the stimuli and refrain from movement as much as possible. To further prevent movement artifacts, the subject’s head was immobilized with padded earmuffs and a foam headrest. Each subject was provided with earplugs to reduce the noise generated by the MRI machine.

#### Psychophysical ratings

Before each functional scanning run, subjects rated pain intensity of the stimuli under 50 ml and 100 ml rectal balloon distention on a 10-point scale. The anchors for pain intensity were between “no pain sensation” and “extremely intense pain sensation”. If the stimulus was rated as a zero on the pain intensity scale, the subject was asked to rate the nonpainful sensation using “no sensation”. If the stimulus was rated as a ten, the subject was asked to rate the maximum sensation using “extreme pressure sensation”. To avoid head movement, all ratings were nonverbal, using the fingers of one hand to indicate perceptual estimates from 0 to 10.

#### Data analysis

BOLD fMRI images were analyzed using statistical parametric mapping 2 (SPM2) (Wellcome Department of Cognitive Neurology, London, UK; http://www.fil.ion.ucl.ac.uk/spm/software/spm2/). Correction for acceptable head movement between the images in each session was performed by alignment with one image. Each subject’s realigned images were resliced to isotropic 2 mm 3 voxels and normalized by linear and nonlinear transformations into a standardized anatomical space (Montreal Neurological Institute). After normalization, a 5 mm (full width half maximum) Gaussian filter was applied to each image. SPM2 treats each voxel according to a general linear model. For each condition, activated and deactivated voxels were identified using an α level of P ≤ 0.05 (uncorrected for multiple comparisons) and used to construct individual statistical parametric maps. All suprathreshold voxels in a statistical parametric map are partitioned into clusters of contiguous (touching) voxels. SPM2 calculates probability values for the spatial extent of each cluster (size in voxels) and the strength of the effect at each individual voxel. The multiple comparisons problem is addressed using continuous random field theory, assuming the statistic image to be a good lattice representation of an underlying continuous stationary random field. This results in inference based on corrected p-values.

## Results

### Subject characteristics

Mean ages (95%CI) did not differ significantly between the moxibustion (47.5 years [41–53]) and control groups (40.9 years [35-47]). Mean weight and height in respective groups were 66 kg (63–69) and 167 cm (162–172) versus 68 kg (62–74) and 169 cm (164–174). The disease course of D-IBS in respective groups was 3 years (2–5) and 3.5 years (3–10)(Table 1).

**Table 1:** **Subject characteristics in both groups**

Item	Moxibustion group (n = 15)		Control group (n = 13)		P
	Mean	95% CI	Mean	95% CI	
Age	47.47 ± 0.896	[41.48, 53.46]	40.92 ± 10.136	[34.8, 47.05]	0.112
Gender	Male 9	--------	Male 7	-------	0.743
	Female 6		Female 6		
Height	1.673 ± 0.886	[1.624, 1.722]	1.692 ± 0.8156	[1.643, 1.742]	0.563
Weight	66.67 ± 5.665	[63.53, 69.80]	68.46 ± 9.70	[62.6, 74.32]	0.565
Course of disease	3.0(2.0-5.0)	[2.26, 8.47]	3.5(3.0-10.0)	[3.39, 8.46]	0.352

### Birmingham IBS symptom scale and IBS QOL scale

In the moxibustion group, the Birmingham IBS Symptom Scale and IBS QOL Scale scores significantly decreased, from 28.27 ± 6.64 to 9.00 ± 4.05 (P < 0.01) and 80.33 ± 8.49 to 48.27 ± 7.69 (P < 0.01), respectively, after treatment. In the control group, the Birmingham IBS Symptom Scale and IBS QOL Scale scores significantly decreased, from 27.38 ± 3.95 to 21.46 ± 4.31 (P < 0.01) and 80.54 ± 8.27 to 70.62 ± 7.42 (P < 0.01), respectively, after treatment. The decreases in the Birmingham IBS Symptom Scale and IBS QOL Scale scores in the moxibustion group was significantly greater than those in the control group (P < 0.01) (Table 2, Table 3).

**Table 2:** **Birmingham IBS symptom scale comparison**

Group	n	Before Treatment	After Treatment	Comparison	
				F	P
Moxibustion Group	15	28.27 ± 6.64	9.00 ± 4.05	417.009	<0.001
Control Group	13	27.38 ± 3.95	21.46 ± 4.31	45.260	<0.001
Total Amount	28	27.86 ± 27.86	14.79 ± 7.54	373.207	<0.001
Comparison between groups	F	0.175	62.040		
	P	0.679	<0.001

**Table 3:** **IBS QOL scale comparison**

Group	n	Before Treatment	After Treatment	Comparison	
				F	P
Moxibustion Group	15	80.33 ± 8.49	48.27 ± 7.69	485.324	<0.001
Control Group	13	80.54 ± 8.27	73.62 ± 7.42	97.200	<0.001
Totally Amount	28	80.43 ± 8.23	60.04 ± 14.86	527.472	<0.001
Comparison between groups	F	0.004	78.191	--	--
	P	0.949	<0.001	--	--

### Rectal distention threshold

Before treatment, the rectal distention thresholds in both groups were not significantly different. After treatment, the first sensation threshold in both groups was not significantly different. The defecation urge threshold of the moxibustion group after treatment was significantly higher than that of baseline (P < 0.01). However, in the control group, there was no significant change after treatment. The pain detection threshold after moxibustion was significantly higher than baseline (P < 0.01). The pain detection threshold increased in the control group but was not statistically significant (Table 4).

**Figure 3:**
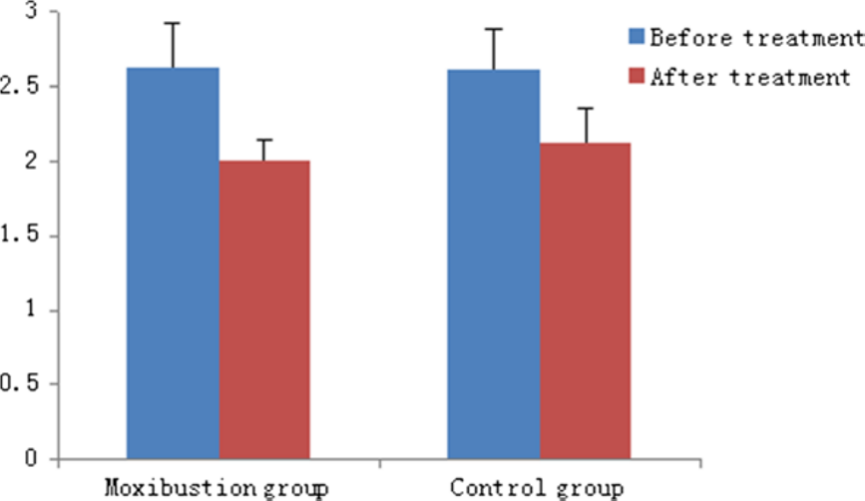
**Pain score in both groups during 50mlrectal balloon distension.**

**Figure 4:**
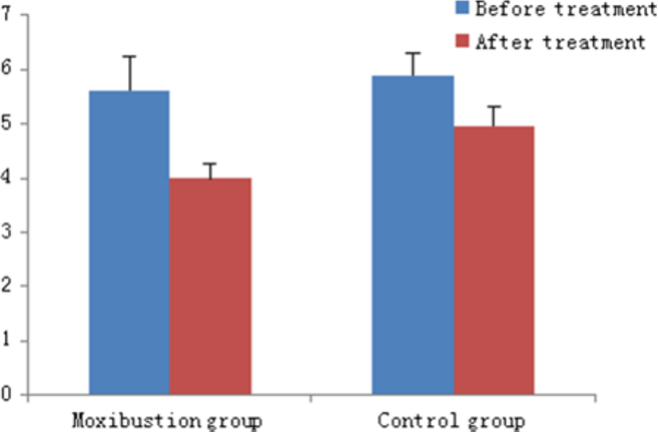
**Pain score in both groups during 100 ml rectal balloon distention.**

The pain score of the moxibustion group during the 50 ml rectal balloon distention decreased from 2.63 ± 0.30 to 2.0 ± 0.15 after treatment (P > 0.05). In the control group, it decreased from 2.62 ± 0.27 to 2.12 ± 0.23 after treatment (P > 0.05). There was no difference between the two groups. The pain score during the 100 ml rectal balloon distention in the moxibustion group after treatment decreased from 5.6 ± 0.63 to 4.0 ± 0.28 (P < 0.05). In the control group, it decreased from 5.89 ± 0.42 to 4.96 ± 0.34 (P > 0.05). The decrease in pain score during the 100 ml rectal balloon distention in the moxibustion group was significantly greater than that of the control group (P < 0.05) (Figure 3, Figure 4).

**Table 4:** **Rectal distention threshold comparison**

Item	First sensation threshold						Defecation urge threshold						Pain detection threshold					
	Before Treatment		After Treatment		Difference		Before Treatment		After Treatment		Difference		Before Treatment		After Treatment		Difference	
	**Mean ± S.E**	**95% CI**	**Mean ± S.E**	**95% CI**	**Mean ± S.E**	**95% CI**	**Mean ± S.E**	**95% CI**	**Mean ± S.E**	**95% CI**	**Mean ± S.E**	**95% CI**	**Mean ± S.E**	**95% CI**	**Mean ± S.E**	**95% CI**	**Mean ± S.E**	**95% CI**
Moxibustion group (n = 15)	22.27 ± 2.052	[21.13,23.40]	22.67 ± 0.63	[21.32,24.0]	0.40 ± 0.81	[−1.34,2.14]	40.07 ± 3.23	[33.13,47.00]	63.47 ± 2.01^▲^	[59.15,67.78]	23.40 ± 2.88^★^	[17.23,29.57]	89.07 ± 5.01	[78.33,99.81]	113.6 ± 7.51^●^	[97.48,129.72]	24.53 ± 5.78^○^	[12.14,36.92]
Control group (n = 13)	22.31 ± 3.351	[20.28,24.33]	21.69 ± 1.17	[19.14,24.2]	−0.62 ± 1.5	[−0.43,2.80]	41.31 ± 1.89	[37.19,45.42]	44.15 ± 1.55	[40.78,47.58]	2.85 ± 1.68	[−0.80,6.50]	89.62 ± 2.85	[83.41,95.82]	93.46 ± 2.67	[87.64,99.28]	3.85 ± 2.84	[−2.33,10.02]

Compared with baseline, ▲ p = 0.000, compared with the control group, ▲ p = 0.000, ★ p = 0.000.

Compared with baseline, ● p = 0.001, compared with the control group, ● p = 0.022, ○ p = 0.005.

### Brain fMRI

There was no brain activation in either group during the 50 ml rectal balloon distention. After treatment, the prefrontal cortex (PFC) was activated in the moxibustion group, while the PFC and anterior cingulated cortex (ACC) were activated in the control group (Table 5).

**Table 5:** **Brain regions significantly activated during 50 ml rectal balloon distention in both groups**

	Moxibustion Group (n = 15)								Control Group (n = 13)							
	Before Treatment				After Treatment				Before Treatment				After Treatment			
	XYZ	BA	T	P	X Y Z	BA	T	P	XYZ	BA	T	P	X Y Z	BA	T	P
Prefrontal Cortex	None				−20 64 -12	----	5.11	0	None				−38 38 -16	----	5.64	0.001
					−20 60 -20	11	4.9	0					−28 28 -20	----	4.13	0.001
					6 36 30	9	4.49	0.004					−36 14 36	9	5.87	0.002
					14 22 60	6	3.56	0.997								
Anterior Cingulate Cortex	None				None				None				−2 -42 30	31	5.36	0.002
													−6 -52 8	30	4.79	0.002

**Figure 5:**
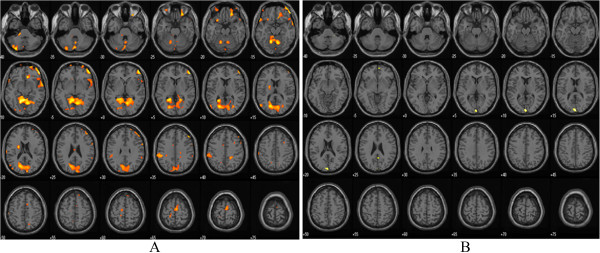
**Functional magnetic resonance imaging in moxibustion group before and after treatment during 100 ml rectal balloon distention: A-before treatment, B-after treatment.**

**Figure 6:**
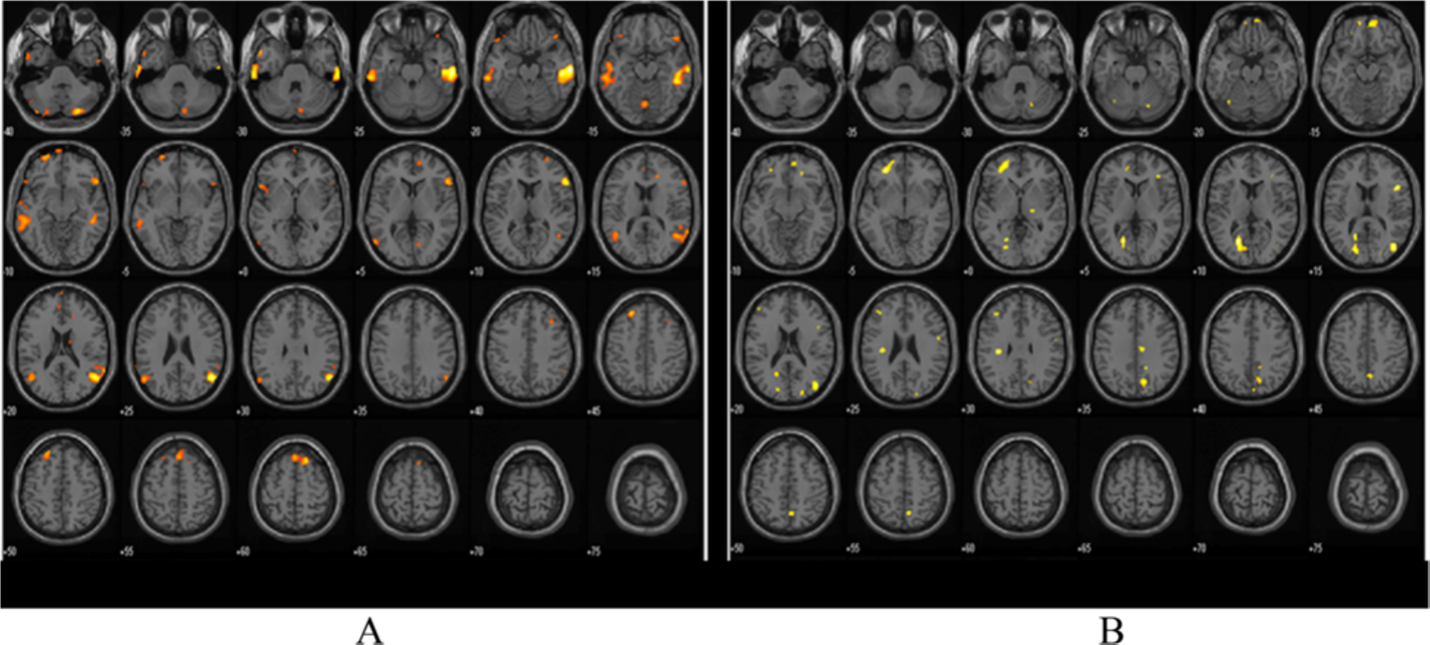
**Functional magnetic resonance imaging chart in control group before and after treatment during 100 ml rectal balloon distention: A-before treatment, B-after treatment.**

**Figure 7:**
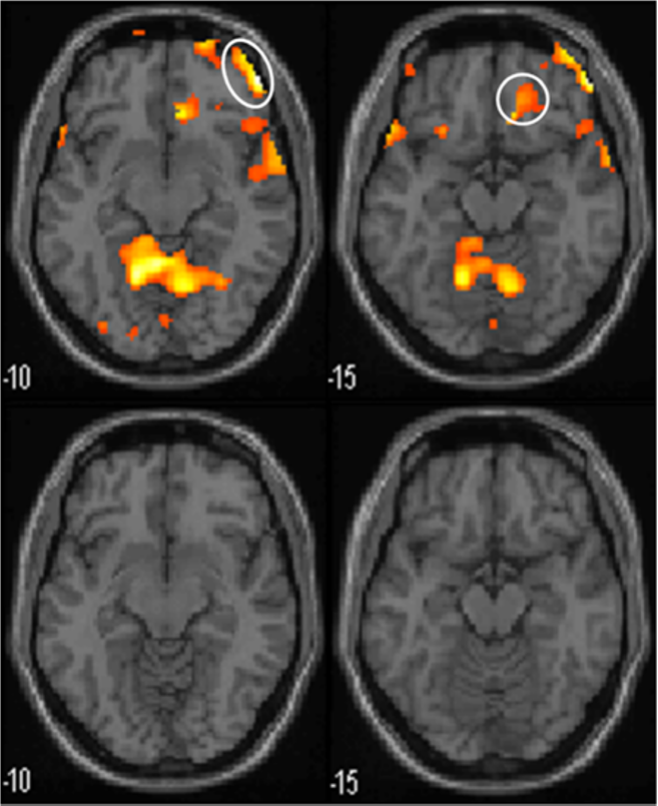
**Functional magnetic resonance imaging in activated PFC and ACC in moxibustion group during 100 ml rectal balloon distention.** Upper row are the pictures before treatment, lower row are the pictures after treatment. The prefrontal cortex(PFC) and anterior cingulated cortex(ACC) are encircled. Left row is PFC and right row is ACC.

**Figure 8:**
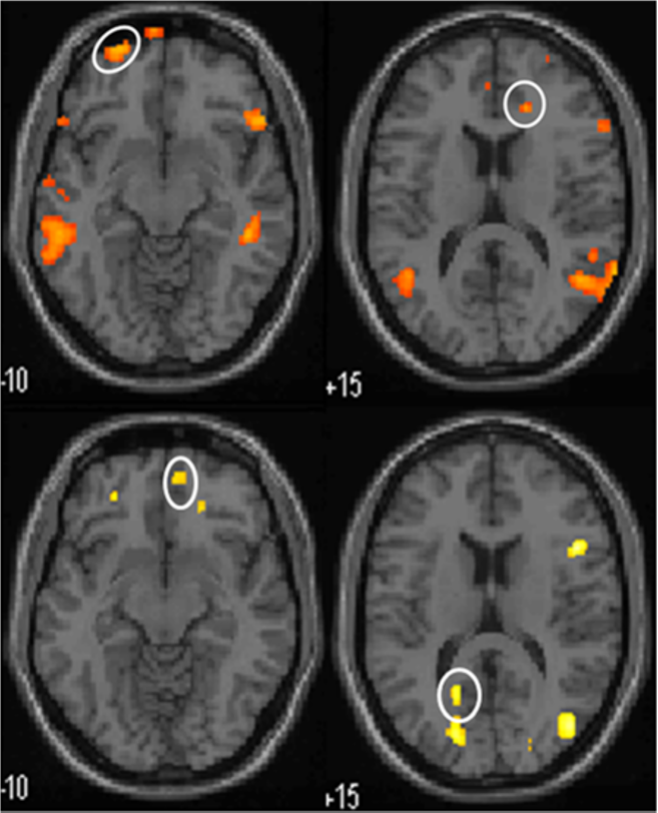
**Functional magnetic resonance imaging of activated PFC and ACC in control group during 100 ml rectal balloon distention.** Upper row is the pictures before treatment, lower row is the pictures after treatment. PFC (left row) and ACC (right row) are encircled.

During the 100 ml rectal balloon distention before treatment, the PFC and ACC were activated. After treatment, there was no activation of the PFC or ACC in the moxibustion group. However, these areas were still activated in the control group, although the specific coordinates of those areas were different from the baseline (Table 6, Figure 5, Figure 6, Figure 7 and Figure 8).

**Table 6:** **Brain regions significantly activated during 100 ml rectal balloon distention in both groups**

	Moxibustion Group (n = 15)								Control Group (n = 13)							
	Before Treatment				After treatment				Before Treatment				After treatment			
Prefrontal Cortex	X Y Z	BA	T	P	XYZ	BA	T	P	X Y Z	BA	T	P	X Y Z	BA	T	P
	26 40 -24	11	4.98	0.000	6 60 -6	10	3.62	0.993	58 24 12	45	6.03	0.001	−24 40 -16	----	3.36	0.024
	26 72 -8	10	5.2	0.000					−24 64 -8	10	5.19	0.006	54 -2 24	6	3.6	0.048
	52 50 -10	47	7.06	0.000					−22 38 46	8	4.95	0.005	−12 46 6	----	3.51	0.05
	52 34 34	9	6.91	0.000					−52 18 0	47	4.16	0.042				
	52 46 40	46	6.31	0.000					−38 34 -14	11	3.74	0.042				
	50 22 -6	47	3.69	0.000												
	−12 -14 60	6	3.86	0.008												
Anterior Cingulate Cortex	10 30 -12	32	5.82	0.000	4 -52 24	31	3.62	0.991	18 34 16	----	4.32	0.049	−20 -66 10	30	4.99	0.001
													10 -20 34	----	3.99	0.042

## Discussion

The increased visceral sensitivity in the pathophysiology of IBS patients has received much attention recently. Our results demonstrate the clinical effects of moxibustion on D-IBS. They also show that the initial perception threshold, urgent defecation perception threshold, and pain perception thresholds of the rectum in two groups of patients before treatment were similar to those in another report [27]. After aconite-separated moxibustion treatment, the defecation urge threshold and pain detection threshold were increased while the pain score after a 100 ml rectal balloon distention was decreased. Therefore, after aconite-separated moxibustion treatment, the rectal sensitivity of D-IBS patients decreased. Moreover, the critical efficacy of aconite-separated moxibustion might be associated with reduced rectal sensitivity.

The development of brain imaging has greatly enhanced the ability to investigate brain–gut interactions and to assess the central nervous system’s role on visceral pain perception. The results of studies using brain imaging in IBS have demonstrated differences in brain activation between patients with IBS and healthy controls [28]. Previous studies have found that IBS patients seem to have different visceral sensory areas from normal controls [29]. Moreover, the blood flow, glucose metabolic rate, or potential activities of the cerebral cortex in patients with IBS are not the same as those in control groups [30, 31]. Studies have shown that anterior cingulate cortex (ACC), PFC, inferior colliculus (IC), and thalamus are activated in the non-painful and painful rectal distension both in IBS and control patients [29]. Results of functional neuroimaging studies in IBS patients show variable results but increased regional activity in the insula (INS) and anterior midcingulate cortex (aMCC) are most commonly reported [32]. Hypersensitive IBS patients had more dorsolateral prefrontal cortex (DLPFC) activation than normosensitive patients [22]. In a study of rectal distension, pain, or discomfort, patients with IBS have fairly variable differences in activated brain regions compared with controls, generally comprising divisions of the PFC, ACC, MCC, insula, amygdala, hypothalamus, and brainstem nuclei [33]. Ran Jun Tao et al. found that the visceral hypersensitivity center of Chinese IBS patients might be the IC and PFC [27]. In our study, for both groups of patients, a significant activation was observed during the 50 and 100 ml rectal balloon distention in the ACC and PFC. Activation was seen in other brain areas, such as temporal lobe, primary visual cortex, cerebellum, but there is no difference between moxibustion group and control group in these areas. They were not examined further in this study.

CH Wilder-Smith et al. [34] showed that brain activation changes during heterotopic stimulation differed highly significantly between constipation predominant IBS(IBS-C), IBS-D, and controls. The main centres affected were the amygdala, anterior cingulate cortex(ACC), hippocampus, insula, periaqueductal gray, and prefrontal cortex(PFC), which form part of the matrix controlling emotional, autonomic, and descending modulatory responses to pain. Winnie CW Chu et al. [35] found that rectal distention induced significant activation of the anterior cingulated cortex(ACC), prefrontal cortex(PFC), thalamus, temporal regions and cerebellum of D-IBS. In our study, after aconite-separated moxibustion treatment, activation of the PFC and ACC during the 100 ml rectal balloon distention was not seen when compared with the control group. Visceral hyperalgesia was accompanied by activation of more PFC areas [36]. Meanwhile, the ACC is considered a key element in the rostral limbic system [37]. Relative to controls, IBS participants showed heightened activation of the ACC, IC, and ventral medial prefrontal regions, suggesting heightened affective responses to painful visceral stimuli [38]. The visceral sensory nerve center contains a regulatory network system, which is composed of the PFC, limbic system including the cingulate gyrus, IC, and thalamus [39]. The formation and sensitivity of the visceral sensory center are closely related to this network system. An altered visceral sensitivity through abnormal endogenous pain processing plays an important role in the pathogenesis of IBS [40]. After aconite-separated moxibustion, the disappearance of PFC and ACC activation may imply that this kind of treatment can decrease affective responses to painful visceral stimulation and down-regulate the influence on the visceral hypersensitivity. This may partially explain a neurobiological mechanism of how aconite-separated moxibustion treatment relieves abdominal pain, bloating, or discomfort in D-IBS patients.

Our study showed that during a 50 ml rectal balloon distention, the activation of the PFC and ACC increased after treatment in both groups. Previous study had shown that the fMRI signal rangeability of insula cortex(IC) and PFC increased with the strength of the rectal balloon stimulus but statistical significance was only found when the distention was above 90 ml [27]. In our study, 50 ml rectal balloon distention is a stimulation under pain threshold, its significance remains further research. The PFC and ACC were both activated before and after placebo moxibustion treatment during the 100 ml rectal balloon distension. Although the activation coordinates were not the same, they all belong to the same anatomical structures. Before and after stimulation, both the ACC and PFC were activated, but the activation of specific parts were subtly different. Whether the response will appear from different brain cell populations at different times for the same stimulation needs further study.

In recent years, many studies have been performed using fMRI to investigate the cerebral matrix related to acupuncture therapy [41, 42]. Acupuncture has a coordinated effect on a network of cortical and subcortical limbic and paralimbic structures in the human brain [43]. Acupuncture also produces extensive deactivation of the limbic-paralimbic-neocortical system [44]. An fMRI study on IBS patients with acupuncture therapy resulted in differential activation of the right insula and pulvinar and medial nucleus of the thalamus after EA treatment [35]. However, there is no brain imaging research on moxibustion.

Moxibustion is an important external treatment used within traditional Chinese medicine that has few side effects. The *Inner Canon of Yellow Emperor Miraculous Pivot* states that, “When needles are not having effect, may moxibustion the appropriate treatment.” In our study, the acupoint combination (ST25, RN6, RN12) was used, and these points were chosen to tonify the spleen and stomach. One recent study of an abdominal acupuncture method including points RN6 and RN12 showed improvement in allometric function of the brain cognition network of the central nervous system [45]. However, whether moxibustion has the same mechanism as needles is still unknown. Because experimental and clinical evidence indicates that most acupuncture effects are mediated by the brain, a brain imaging study on moxibustion should be informative. Previous acupuncture fMRI studies have mostly investigated the effects of stimulating one or two acupoints simultaneously [46]. However, we observed comparison imaging of D-IBS patients before and after consecutive moxibustion treatment sessions.

This study has some potential weaknesses. First, the sample size is limited. The current data need to be confirmed with a larger patient population. Second, manual volume-based distensions rather than barostat pressure controlled inflations were used because of equipment limitations. In further studies, a barostat pressure measurement should improve the accuracy of the experiment. Third, the definite activity of neurons in the PFC and ACC are not known. We can only reveal the mechanism of aconite-separated moxibustion using fMRI. With more standardized IBS distension protocols and advanced imaging techniques [47] we could further understand the mechanisms of moxibustion for D-IBS patients.

## Conclusions

Moxibustion can improve the symptoms and quality of life in D-IBS patients and decrease rectal sensitivity. The mechanism may involve the regulation of abnormal endogenous pain processing in D-IBS. Our results provide support of the clinical use of moxibustion for the treatment of D-IBS.

## Abbreviations

D-IBS: Diarrhea type of irritable bowel syndrome
fMRI: Functional magnetic resonance imaging
QOL: Quality of life
PFC: Prefrontal cortex
ACC: Anterior cingulated cortex
IBS-C: Constipation type of Irritable bowel snydrome
IC: Insula cortex.
